# Quantitative dynamic granzyme B PET imaging to characterize novel combination immunotherapy response in preclinical glioblastoma models

**DOI:** 10.7150/thno.123588

**Published:** 2026-01-01

**Authors:** Carlos A. Gallegos, Yun Lu, Patrick N. Song, Rodrigo Queiroz, Ugur Akca, Sharmila Sridhar, Jennifer L. Bartels, Yujun Zhang, Li Li, Jason M. Warram, Suzanne E. Lapi, Benjamin M. Larimer, James M. Markert, Anna G. Sorace

**Affiliations:** 1 Department of Biomedical Engineering, University of Alabama at Birmingham, Birmingham, AL.; 2 Department of Radiology, University of Alabama at Birmingham, Birmingham, AL.; 3 Graduate Biomedical Sciences, University of Alabama at Birmingham, Birmingham, AL.; 4 Department of Neurosurgery, University of Alabama at Birmingham, Birmingham, AL.; 5 Department of Otolaryngology, University of Alabama at Birmingham, Birmingham, AL.; 6 Department of Chemistry, University of Alabama at Birmingham, Birmingham, AL.; 7 O'Neal Comprehensive Cancer Center, University of Alabama at Birmingham, Birmingham, AL.

**Keywords:** glioblastoma, oHSV, immunotherapy, dynamic PET, compartment modeling

## Abstract

**Rationale:** Immune-targeted positron emission tomography (PET) allows for the non-invasive monitoring of immune populations with potential to provide early markers of response to novel immunotherapies in glioblastoma (GBM). Dynamic PET acquisitions can inform on tracer kinetics via dynamic modeling to enhance the GBM tumor microenvironment characterization, while its impact has yet to be understood. The objective of this study is to evaluate the quantitative description of dynamic granzyme B (GZP)-PET imaging and its ability to inform on combination immunotherapy response in orthotopic syngeneic GBM models.

**Methods:** Orthotopic GBM murine models (GSC005-luc) were imaged with dynamic [^64^Cu]-NOTA-GZP PET (0-80 min) and *T_2_*-weighted MRI one week post-treatment with saline or combination M002 virotherapy and anti-PD1 immunotherapy. One-, two-, and three-tissue compartment models were evaluated for suitability to tracer kinetics via Akaike information criterion. Biological validation consisted of *ex vivo* brain PET imaging, autoradiography, H&E and immunofluorescence for granzyme B. A subset of mice was longitudinally monitored via dynamic [^64^Cu]-NOTA-GZP PET at days 4 and 7. Changes in viable cell bioluminescence and radiological tumor volume (MRI) were used to determine response. Imaging-derived metrics including *k_1_*, *k_2_*, *k_3_, k_4_* and SUV were evaluated across treatment and response groups via unpaired two-tailed T test.

**Results:** Intratumoral [^64^Cu]-NOTA-GZP PET tracer kinetics showed improved fitting based on a two-tissue compartment model (p < 0.05). Intratumoral effector cell function (SUV_mean_ TBR) and tracer binding rate (*k_3_*) were positively correlated with histological granzyme B density (p < 0.01). Increases in tumor tracer influx (*k_1_*) were observed in responders relative to non-responders (p < 0.01).

**Conclusions:** Mathematical description of tracer kinetics via dynamic [^64^Cu]-NOTA-GZP PET offers complementary quantitative metrics for the characterization of immunotherapy response in GBM. Integration of dynamic protocols to immune-target PET approaches can provide clinically translatable metrics to differentiate immunotherapy-induced effects from tumor progression in GBM.

## Introduction

Molecular imaging through positron emission tomography (PET) allows for the non-invasive monitoring of metabolic and functional processes by tracking metabolites, proteins and cell surface receptors 1. [^18^F]-fluorodeoxyglucose ([^18^F]FDG) PET, which relies on radiolabeled glucose to evaluate changes in tumor metabolism, serves as the primary molecular imaging approach for diagnosis, monitoring and prognosis in clinical oncology 2. However, this modality has limited utility in neuro-oncology and immunotherapy response assessment given the natural high metabolic activity in normal brain tissue and activated inflammatory cells, resulting in decreased specificity and prognostic potential for brain malignancies such as glioblastoma multiforme (GBM) 1,3. Non-invasive monitoring of immune cell populations via immune-targeted PET imaging offers unique spatiotemporal insights into immunotherapy-induced immune kinetics with potential to identify markers of early therapeutic response in GBM.

Immunotherapies utilizing oncolytic viruses have shown promise in the management of GBM by stimulating innate and adaptive immune responses through the release of immune-promoting signals and tumor antigens following cancer cell lysis 4,5. In particular, virotherapy using M002 oncolytic herpes simplex virus (oHSV), which carries a therapeutic payload of the proinflammatory cytokine interleukin (IL)-12, has shown enhanced promotion of immunological responses and prolonged survival in preclinical GBM models 6,7. Additional preclinical studies have highlighted the improved immunological and survival benefit of combination oncolytic virotherapy with immune checkpoint blockade (ICB) therapies such as those targeting the programmed cell death 1 (anti-PD1) receptor 8,9. This potential for improved therapeutic outcomes of M002 and its combination with anti-PD1 immunotherapy has led to Phase I/II clinical trials of M032, the human equivalent of M002, as single agent (NCT02062827) and combination immunotherapy (NCT05084430) for recurrent or progressive high-grade gliomas 7. Clinical trials for M032 and other oHSV-based therapies have highlighted the limitations of conventional anatomical magnetic resonance imaging (MRI) in differentiating early progression from therapy-induced effects which include immune infiltration and localized inflammation 10-12. These findings underscore the need for novel non-invasive approaches to characterize immune response kinetics following immunotherapy to allow for the early identification of therapeutic benefit and further optimize clinical interventions in GBM management.

In our previous study, we demonstrated that increased spatial homogeneity in intratumoral CD8^+^ immune cell distribution was associated with improved immunotherapy response in preclinical GBM models 9. Of importance, this study also showed a mismatch between CD8^+^ cell infiltration and therapeutic efficacy, potentially attributed to the presence of immune inactivation or exhaustion within the tumor microenvironment. This discrepancy highlighted the importance of exploring additional biomarkers indicative of downstream cytotoxic activity. In this context, granzyme B (GZB), a serine protease released by activated CD8^+^ T cells and natural killer (NK) cells upon recognition of target cells, has been highlighted due to its role in inducing apoptotic pathways in virally infected or malignant cells through activation of caspase pathways 13. Non-invasive imaging of this effector molecule, via GZB specific PET imaging agent (GZP) PET, has been shown to be predictive of ICB immunotherapy outcomes in preclinical colon and triple negative breast cancer models 14,15. This preclinical evidence has allowed for the development of a Phase I clinical trial to evaluate the safety and effectiveness of GZP-PET in cancer immunotherapy (NTC04169321). Despite this potential, there have been limited studies focusing on its utility on oHSV response in GBM and whether complementary kinetic imaging-derived metrics can be used to extract additional biologically relevant information.

Integration of dynamic PET protocols allows for the characterization of tracer pharmacokinetics via compartment modeling, which describes intratumoral tracer states associated with vascular perfusion and binding potential. These models allow for the derivation of quantitative metrics for tracer influx (*k_1_*) and efflux (*k_2_*), related to vascular access, and tracer binding (*k_3_*) and unbinding (*k_4_*), informative on tracer-target interactions, as shown in preclinical studies using amino acid-based PET tracers 16. These quantitative metrics derived from PET tracers descriptive of tumor viability and amino acid transport have been shown to have diagnostic value and correlate with patient survival in clinical malignant brain tumor studies when treated with standard of care therapies 17,18. Nonetheless, the use of compartment modeling to describe immune-target PET tracer kinetics and its potential to enhance immune-targeted PET assessment for GBM immunotherapy response remain unexplored.

In this contribution, we developed and optimized a compartment model descriptive of dynamic [^64^Cu]-NOTA-GZP PET imaging to quantify vascular delivery, tracer retention, and effector cell function following combination M002 oHSV and anti-PD1 ICB immunotherapy in an orthotopic GBM model. In addition, we longitudinally assessed dynamic [^64^Cu]-NOTA-GZP PET-derived metrics and their association with immunotherapy response. To our knowledge, dynamic [^64^Cu]-NOTA-GZP PET has not been previously evaluated for describing immune-target PET tracer kinetics and informing on immunotherapy-induced changes in preclinical GBM models. For our short-term assessment, an orthotopic GSC005-luc GBM model was imaged using anatomical *T_2_*-weighted MRI, followed by dynamic [^64^Cu]-NOTA-GZP PET to evaluate early vascular and effector cell function changes following combination immunotherapy. Imaging findings were further validated via *ex vivo* brain PET imaging, autoradiography and immunofluorescence (IF) for GZB. An additional longitudinal cohort underwent serial dynamic [^64^Cu]-NOTA-GZP PET and anatomical MRI and was monitored for long-term survival for metric comparisons based on immunotherapy response. Non-invasive assessment of vascular properties and effector cell function can provide clinically translatable metrics to monitor therapeutic efficacy and predict survival following novel combination immunotherapies in GBM.

## Materials and Methods

### Short-term model development and validation

Murine GBM models treated with combination immunotherapy or saline controls were imaged using dynamic [^64^Cu]-NOTA-GZP PET/CT one week following treatment for the comparative assessment of one-, two- and three tissue compartment models. The optimal model was compared with *ex vivo* PET, autoradiography, and histological analysis for biological validation.

#### GBM model development, growth monitoring and treatment

**Figure 1:**
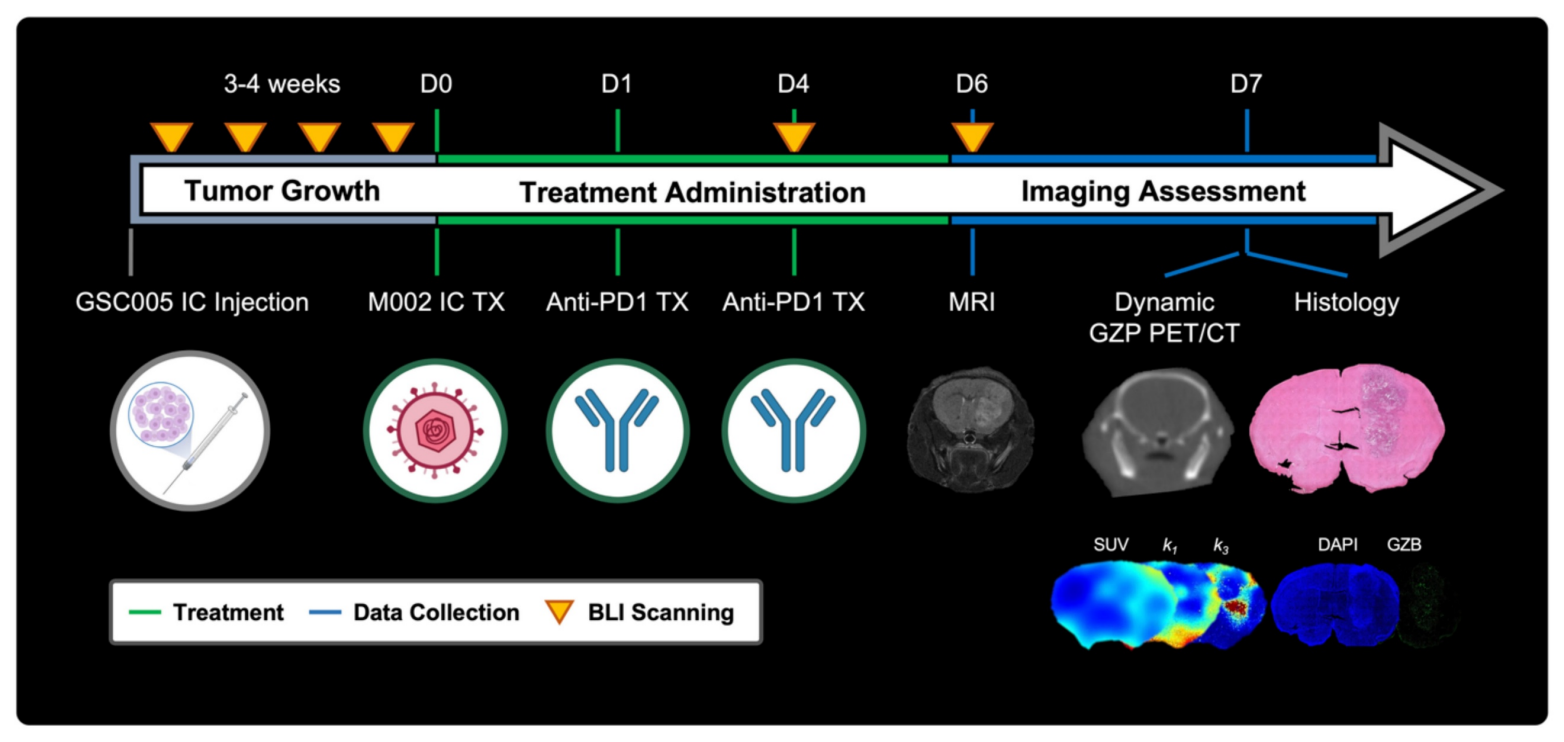
** Representative schematic for short-term dynamic [^64^Cu]-NOTA-GZP PET imaging assessment.** Experimental timeline including key timepoints for tumor growth monitoring, treatment administration, and imaging assessment with representative schematics. IC - intracranial, TX - treatment, SUV - standardized uptake value, GZB - granzyme B.

All animal experiments were approved by the Institutional Animal Care and Use Committee (IACUC, protocol 21611) at the University of Alabama at Birmingham (UAB). Orthotopic syngeneic GBM murine models were developed and treated as previously described with minor modifications 9. In brief, 6-8-week-old C57BL/6 (Charles River Laboratories, Wilmington, MA) were intracranially implanted with 5 × 10^5^ GSC005-luc^+^ cells on the right brain hemisphere. Subsequently, tumor growth was monitored via weekly bioluminescence imaging (BLI) (IVIS Lumina III, Perkin Elmer, Walthan, MA), acquired 10 min following intraperitoneal administration of D-luciferin (115144-35-9, GoldBio, Olivette, MO). Quantification of total flux (p/s) was performed utilizing a standardized circular region of interest (ROI) placed on the cranial region using Living Image (Revvity, Waltham, MA); a tenfold increase from background signal was selected as threshold for enrollment. Once enrolled, mice were randomly assorted into two treatment groups: combination M002 and anti-PD1 immunotherapy (N = 27) or saline control (N = 12) groups. Combination immunotherapy mice received an intratumoral injection of M002 (1 × 10^7^ pfu/ 5 ul) on day 0, followed two intraperitoneal doses of anti-mouse PD1 (RMP1-14, Bio X Cell, Lebanon, NH) at 10 mg / kg on days 1 and 4. See **Figure 1** for experimental timeline.

#### Anatomical MRI protocol and analysis

Anatomical MRI scans were collected using a 9.4 T preclinical scanner (Bruker Biospect, Billerica, MA) to confirm tumor presence prior to treatment and delineate tumor boundaries for intratumoral dynamic [^64^Cu]-NOTA-GZP PET assessment post-therapy. Acquisition consisted of a high-resolution axial *T_2_*-weighted scan collected using a rapid acquisition with refocusing echoes (RARE) sequence with an echo time (TE) of 40 ms, repetition time (TR) of 2500 ms, flip angle (FA) of 180º, and voxel size of 0.08 × 0.08 × 0.5 mm^3^ in a 256 × 256 acquisition matrix with 21-23 slices. Tumor region was manually delineated based on *T_2_* signal hyperintensity using VivoQuant (v.2022, Invicro, Needham, MA). In addition, a contralateral normal brain circular ROI was manually placed on three slices where viable tumor was observed for normalization of quantitative metrics.

#### [^64^Cu]-NOTA-GZP radiolabeling

NOTA-Ala-Gly-Gly-Ile-Glu-Phe-Asp-CHO (NOTA-GZP) was radiolabeled with ^64^Cu, provided by UAB cyclotron, based on previously described approaches with slight modifications 19. The ^64^CuCl_2_ / HCl solution was equilibrated to a pH of 6 using 1 M sodium acetate buffer (229873, Millipore Sigma, Burlington, MA) and mixed with 50 μL of NOTA-GZP (1 μg / μL). The reaction was carried out by heating the mixture at 37 ºC, 600 rpm, for 45 min. The final solution was loaded into a C18 Sep-Pak column (WAT020515, Waters, Milford, MA) and eluted using 200 μL of 70% EtOH in PBS, followed by 200 μL of PBS. Radiotracer purity was assessed using high-performance liquid chromatography (HPLC), where a purity > 95% was defined as the threshold to use for imaging. Of importance, previous studies have utilized ^68^Ga for the purposes of GZP radiolabeling 19-21. In this study, ^64^Cu was selected based on the reported improvements in spatial resolution and longer half-life 22, allowing for improved characterization of tracer dynamics and exploration of imaging-based validation via *ex vivo* PET and autoradiography.

#### Dynamic [^64^Cu]-NOTA-GZP PET/CT imaging acquisition and analysis

Dynamic [^64^Cu]-NOTA-GZP PET was collected using a preclinical small animal PET/CT imaging system (GNEXT, Sofie Bioscience, Culver City, CA) seven days from the start of therapy. Scan consisted of an 80-min list-mode acquisition, with 100 ± 10 μCi (3.7 ± 0.37 MBq) of [^64^Cu]-NOTA-GZP being administered via tail-vein catheter followed by a saline flush. Subsequently, a 5-min computed tomography (CT, voltage, 80 kVp; current, 150 μA) scan was collected for anatomical reference. A subset of mice (N = 5 treatment, 3 controls) was humanely euthanized, and brains were excised for a 1 h *ex vivo* PET/CT brain scan and, upon completion, placed in formalin and fixed overnight. Remaining mice were monitored longitudinally for survival.

**Figure 2:**
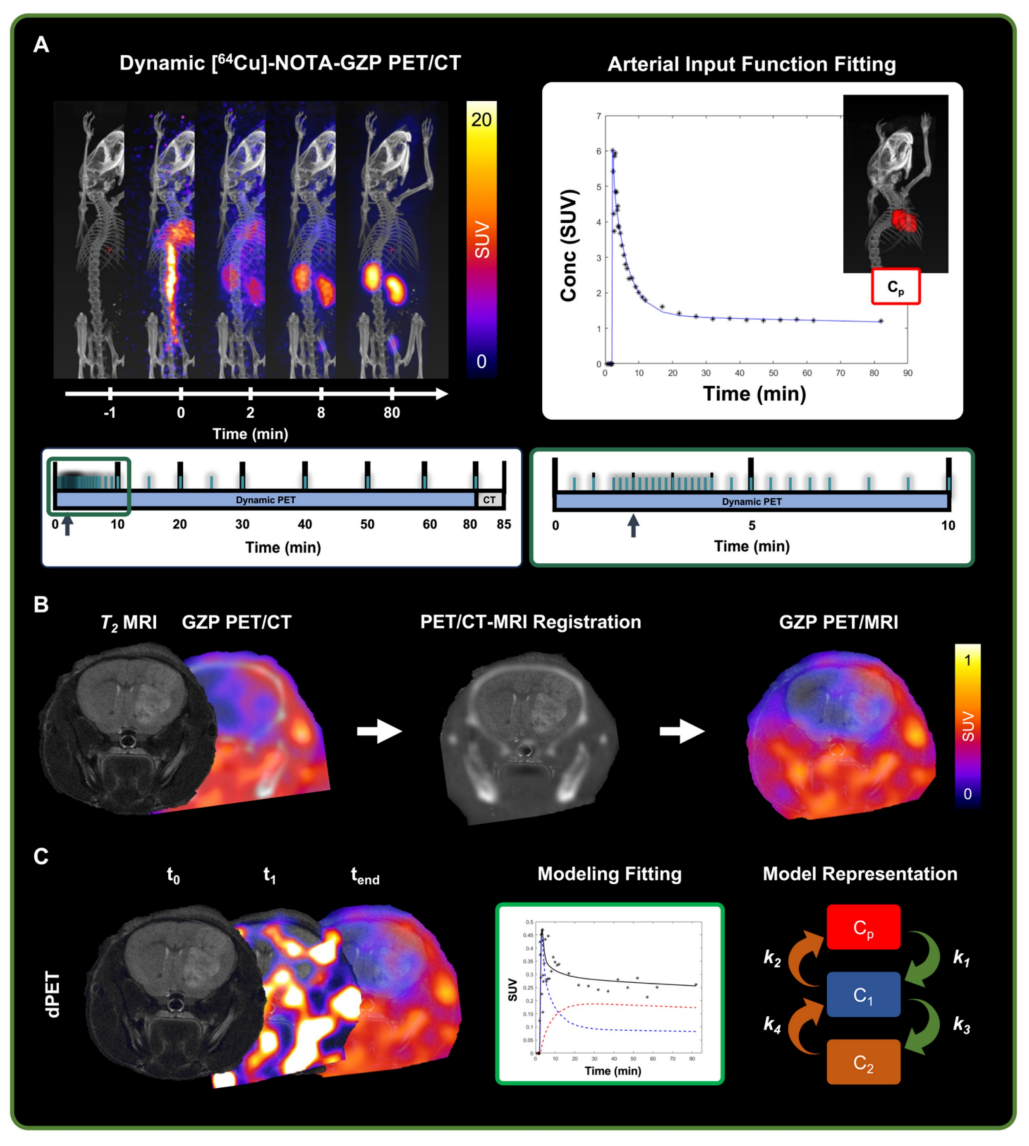
** Representative schematic for dynamic [^64^Cu]-NOTA-GZP-PET processing. A)** Representative scans from dynamic [^64^Cu]-NOTA-GZP PET/CT acquisition (top left) with acquisition time sequence (bottom), blue lines represent generated imaging frames. Arterial input function, collected from a manually drawn heart ROI, was fitted using a triple exponential function (top right). **B)** Representative schematic for PET/CT and *T_2_* MRI scans, which were manually registered for the assessment of intratumoral tracer uptake. **C)** Representative timepoints for dynamic [^64^Cu]-NOTA-GZP PET acquisition (left), where tracer accumulation can be seen within the tumor region. Mathematical models describing tracer kinetics were fitted to the tumor time activity curves (middle) based on the definition of various intratumoral states (right) including intravascular (C_p_), and intratumoral free (C_1_) and bound (C_2_).

Dynamic PET data was reconstructed using a 3D ordered-subset expectation maximization algorithm (24 subsets, 3 iterations) with a reconstructed voxel size of 0.5 × 0.5 × 0.5 mm^3^ based on a 12 × 10 s, 6 × 30 s, 5 × 60 s, 10 × 300 s, 1 × 1200 s framing sequence (**Figure 2A**) 16. PET sequences were rigidly registered to the reference anatomical CT scan. Voxel-wise conversion to standardized uptake value (SUV) was then performed based on the following equation:


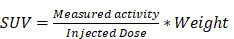

(1)

where measured activity is defined as the quantified uptake within a region in becquerels (Bq), injected dose is the total decay corrected dose administered to the mouse in Bq, and weight is the mouse weight collected at the time of scan collection in grams (g). Subsequently, head region was segmented using VivoQuant's Brain Atlas Tool and manually registered to the *T_2_*-weighted anatomical MRI using anatomical landmarks based on previously described methods (**Figure 2B, Figure S1**) 23,24.

#### Dynamic [^64^Cu]-NOTA-GZP PET mathematical model evaluation

For pharmacokinetic modeling, an image-derived arterial input function (AIF) was collected from a manually drawn ROI in the heart. Based on the results of an AIF fitting comparative assessment of various mathematical models (**Figure S2**) 25-27, dynamic heart time activity curves were fitted to the following multi-exponential model:


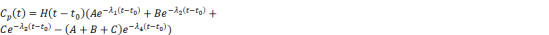

(2)

where C_p_(t) represents the plasma concentration as a function of time, and t_0_ denotes the initial time of tracer injection. Of note, for this assessment, plasma and whole blood were assumed to be equivalent for the quantified cardiac ROI to measure the AIF. The parameters A, B, and C account for changes in magnitude of the exponentials and were bounded to a range of 0 - 100, while λ_1_, λ_2_, λ_3_, λ_4_ are the corresponding rate constants restricted to a range of 0 - 5 for λ_1_, λ_2_, λ_3_ and 0 - 10 for λ_4_ 27.

A comparative assessment of one-tissue, two-tissue and three-tissue compartment models (1TCM, 2TCM, 3TCM) was performed to determine their suitability in describing expected tracer kinetics (**Figure 2C, 3A-B**). These models integrate compartments to account for distinct intratumoral [^64^Cu]-NOTA-GZP tracer patterns, including free or unbound (C_1_), specifically bound to GZB (C_2_) and non-specifically bound to or retained in other molecular structures (C_3_) 28. Mathematical fitting was performed using nonlinear least squares based on the following derived ordinary differential equations:


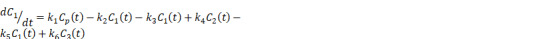

(3)


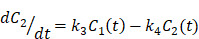

(4)


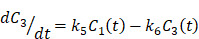

(5)

where *k_1-6_* represent [^64^Cu]-NOTA-GZP transfer rate from plasma to tumor (*k_1_*), tumor to plasma (*k_2_*), GZB binding (*k_3_*) and unbinding (*k_4_*), and nonspecific binding (*k_5_*) and unbinding (*k_6_*). For a 1TCM, k_3-6_ are set to zero, effectively simplifying the model to consider intratumoral free and bound tracer as similar states 28. Similarly, for a 2TCM, *k_5_* and *k_6_* are set to zero, reducing the model to solely account for free and bound tracer states. In 3TCMs, all tracer rates are fitted, providing the most comprehensive assessment of potential tracer states.

The Akaike information criterion (AIC) was used as metrics to select the most suitable model 29. The mathematical expression for this metrics goes as follows:


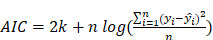

(6)

where *y* represents the measured average tumor time activity curve values, 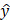
is the predicted values derived from the model, n is the number of timepoints (N = 40 frames), and *k* is the total number of parameters in the model (1TCM = 2, 2TCM = 4, 3TCM = 6). The model with the lowest AIC was deemed the most suitable for this tracer's kinetics. In addition, quantitative metrics including SUV_mean_, defined as the average SUV of all the voxels within the tumor ROI at the final 20 min frame (60-80 min) allowing for the standard circulation time of 60 min following tracer injection, *k_1_* and *k_2_* were evaluated to assess similarities across modeling approaches.

### Biological validation assays

#### Autoradiography acquisition and processing

Autoradiography on *ex vivo* brain tissues was performed following overnight formalin fixation as previously described 9. In short, 1 mm coronal brain sections, sliced using a rodent brain matrix (Braintree Scientific, Braintree, MA), were placed on a exposure cassette (GE Healthcare, Chicago, IL) for 3 h along with three 2 μL standards of known activity (37, 74 and 370 Bq). Autoradiography scans were collected using a laser-scanner (Amersham Typhoon, Cytiva, Little Chalfont, UK) with a pixel size of 50 μm. Quantification consisted of signal calibration (VivoQuant's Autoradiography calibration tool), followed by brain segmentation using optimal thresholding. Upon segmentation mean, max and peak signal concentration (Conc) was calculated for individual brain slices.

#### Histology staining and quantification

Two brain sections, selected based on highest autoradiography signal, were paraffin embedded and sliced into 5 μm sections for histological analysis. Hematoxylin and eosin (H&E) procedures were performed based on past protocols by the UAB pathology core facility 9,30. IF staining for GZB followed a previously published protocol with some modifications 31. Slides were dewaxed and rehydrated with xylene and ethanol, underwent antigen retrieval with citrate buffer, and blocked and permeabilized with 1% BSA and 0.05% Triton 100 in PBS for 30 min at room temperature. Incubation with a primary goat anti-GZB antibody (1:50, AF1865, Bio-Techne Corporation, Minneapolis, MN) was performed overnight at 4 ºC. Subsequently, incubation with secondary antibody DyLight 755 donkey anti-goat (1:200, SA5-10091, ThermoFisher Scientific, Waltham, MA) was performed for 1 h at room temperature in the dark. Finally, slides were mounted with DAPI-containing mounting media (0100-20, SouthernBiotech, Birmingham, AL) and allowed to dry overnight. H&E and IF stained brain sections were then scanned at 20x magnification using a dedicated slide scanner (EVOS M700, ThermoFisher Scientific).

Quantification of H&E and IF slides was performed using a custom QuPath (v.0.5.1, Edinburg, United Kingdom) workflow 32. Semi-automated H&E tumor region segmentation was performed based on a region of increased cellular density with a threshold of 750 cells per mm^2^, followed by manual corrections for tumor boundaries and misclassified regions. Tumor fraction was defined as total tumor area from H&E divided by total brain area. Similarly, IF tumor region was defined based on cellular density metrics obtained from Stardist cell detection on the DAPI channel 33, followed by manual corrections based on the H&E defined region. Positive GZB was defined based on singular objects segmented with a manually defined threshold uniformly implemented across all samples. GZB density was then calculated as the average number of GZB elements divided by the total tumor area in mm^2^, averaged over the two tissue sections collected from each brain.

### Long-term survival assessment

GSC005-luc orthotopic mouse models were developed and treated with single dose M002 followed by a dose of anti-PD1 on days 1, 4 and 7 (N = 22), or saline at similar timepoints (N = 9). 80-min dynamic [^64^Cu]-NOTA-GZP PET/CT scans were performed at day 4 and 7 following treatment, with tumor and contralateral brain time activity curves being fitted to a 2TCM as selected based on prior comparative assessment. Metrics for average SUV, *k_1_*, *k_2_*, *k_3_* and *k_4_* were quantified on the tumor region with additional normalization to contralateral healthy brain (TBR).

In addition, anatomical MRI scans were collected prior to the start of treatment (D-1), and at days 3, 6 and 13 following treatment for anatomical PET/CT reference and monitoring of tumor volumes. Longitudinal monitoring was performed via BLI every three days. Mice were humanely euthanized upon exhibiting signs of neurological decline or 20% body weight loss from the start of treatment. Response was defined based on a threshold of less than 20% increase in BLI signal one month following initial immunotherapy administration as previously reported 9.

### Statistical analysis

**Figure 3:**
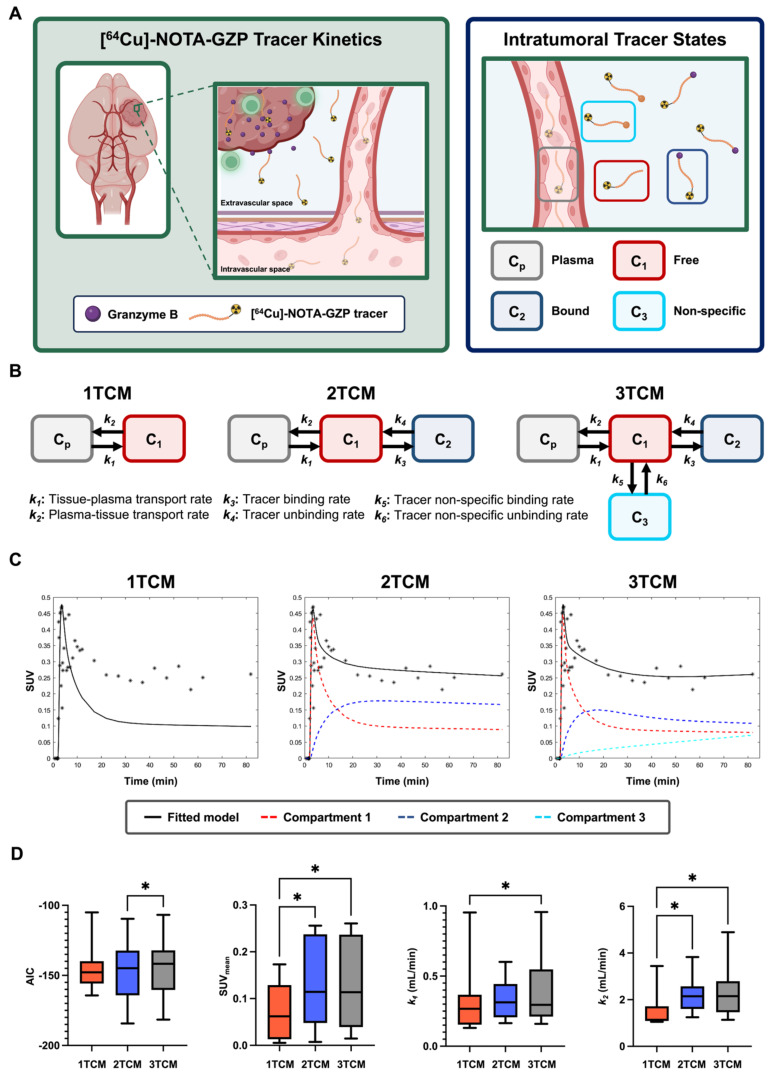
** Dynamic [^64^Cu]-NOTA-GZP PET compartment model comparison revealed improved quality and comparable quantitative metrics with a two-tissue compartment model. A)** Representative schematic for considered tracer dynamics with circulation from vasculature into tumoral space, where specific binding occurs with granzyme B released by cytotoxic immune populations (left). Modeling was performed based on three primary tracer states: free or unbound, specifically bound to granzyme B, and non-specific binding to alternative targets (right). **B)** Representative diagrams for one-, two- and three-tissue compartment models with representation of expected tracer rates by each modeling approach. **C)** Representative fitting for a tumor dynamic [^64^Cu]-NOTA-GZP PET acquisition for each model with the inclusion of individual compartments. **D)** Evaluation of AIC revealed improved fitting, as shown by decreased AIC, with 2TCM when compared to 3TCM (*, p < 0.05, one-way RM ANOVA). Quantitative metrics for SUV and *k_2_* were significantly lower when utilizing a 1TCM relative to 2TCM and 3TCM (*, p < 0.05), while no significant differences were seen across metrics between 2TCM and 3TCM.

Statistical evaluations were performed using Prism 10 (v10.4.1, GraphPad Software, Boston, MA). Comparisons across mathematical model-derived metrics were performed using a one-way repeated measures (RM) ANOVA with Geisser-Greenhouse and Tukey corrections. Furthermore, comparisons between tumor and contralateral brain were performed using a paired two-tail T test, while evaluations between treatment and response groups were performed using unpaired two-tailed T tests. Pearson's correlation tests were further conducted to determine associations of imaging findings and histological quantification. Finally, statistical outliers (N = 1, Figure 3D; N = 4, Figure 3C middle; N = 5 Figure 3C right; N = 1, Figure 5D left; N = 3, Figure 5D right) were identified and excluded using Grubb's test for outliers (α = 0.05).

## Results

### Quantitative comparisons across compartment models revealed improved [^64^Cu]-NOTA-GZP tracer kinetic description with a 2TCM

AIC, SUV, *k_1_* and *k_2_* metrics were compared across 1TCM, 2TCM, and 3TCM fittings for tumor [^64^Cu]-NOTA-GZP PET tracer kinetics (**Figure 3C**). Paired comparisons revealed significant decreases in AIC when utilizing a 2TCM (-146.9 ± 23.31) when compared to a 3TCM (-144.2 ± 22.68, p < 0.05), while no significant differences were observed between these models and 1TCM (-144.4 ± 16.96, p > 0.05), see **Figure 3D**. Quantification of SUV_mean_, as defined by the final timepoint signal (60-80 min), revealed significant decreases when utilizing a 1TCM (0.07 ± 0.06) compared to 2TCM (0.13 ± 0.09, p < 0.05) and 3TCM (0.13 ± 0.10, p < 0.05), potentially attributed to the absence of tracer binding properties associated with PET tracers. Similarly, significant decreases in *k_1_* and *k_2_* were observed in 1TCM (*k_1_*, 0.31 ± 0.26; *k_2_*, 1.51 ± 0.82) fittings when compared to 3TCM (*k_1_*, 0.39 ± 0.26; *k_2_*, 2.32 ± 1.19; p < 0.05), with a comparative decease in *k_2_* only relative to 2TCM (2.29 ± 0.80, p < 0.05). No significant differences were found across these metrics in 2TCM and 3TCM results (p > 0.05). The 2TCM model was selected as the most suitable for the description of this tracer kinetics based on its lower AIC compared to 1TCM and 3TCM approaches. Nonetheless, despite quantified statistically significant differences, AIC values between 2TCM and 3TCM are comparable, where both models provide an appropriate characterization of tracer kinetics, while a 2TCM remains a more parsimonious approach.

### *Ex vivo* and histological analysis showed correlation with dynamic [^64^Cu]-NOTA-GZP imaging metrics

**Figure 4:**
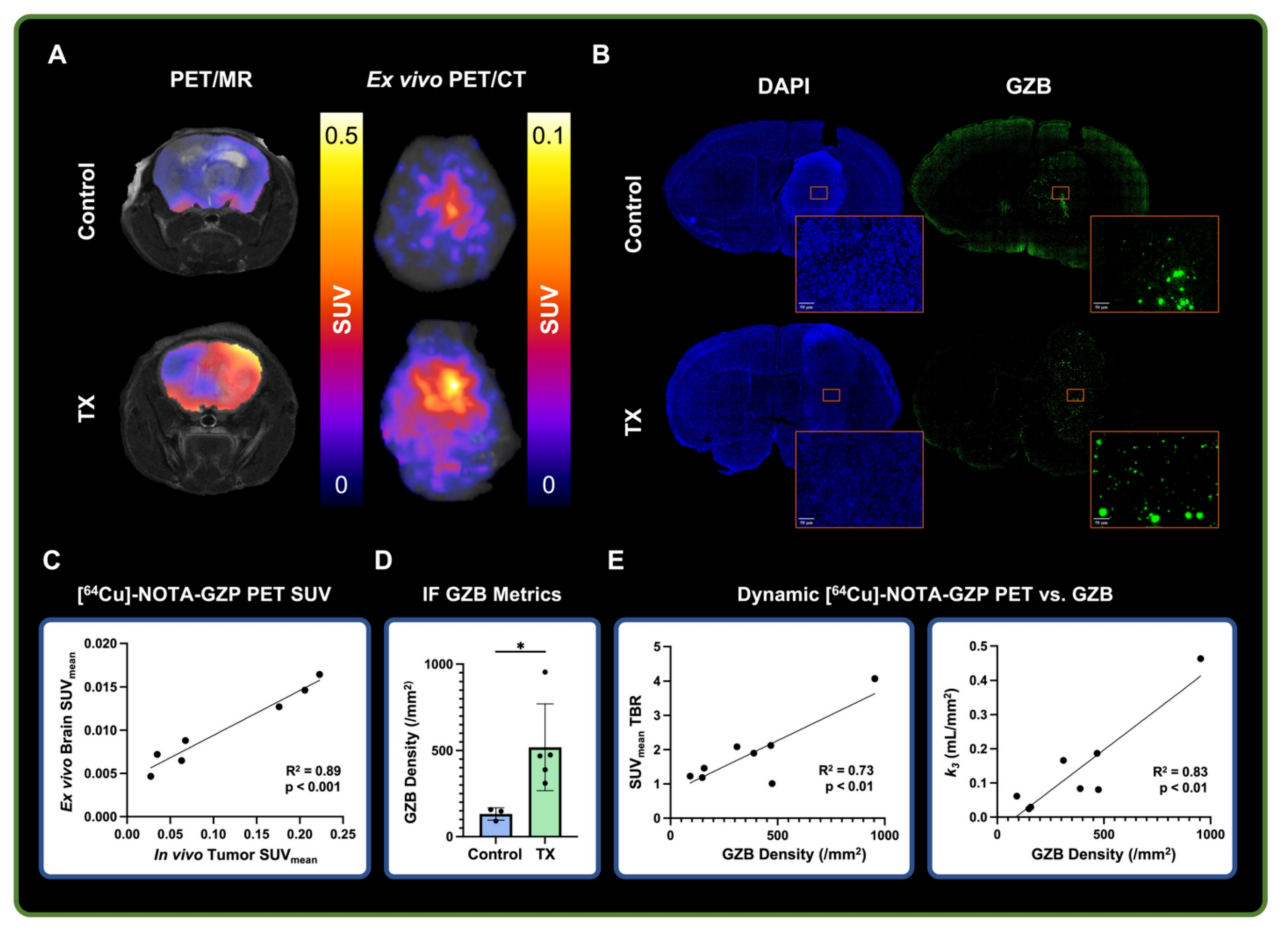
***Ex vivo* and histological findings were associated with dynamic [^64^Cu]-NOTA-GZP PET imaging metrics. A)** Representative *in vivo* [^64^Cu]-NOTA-GZP PET/MRI and *ex vivo* PET/CT scans for control and combination M002 and anti-PD1 treatment groups. **B)** Representative granzyme B (GZB) IF scan for control and treated brain samples, with magnified intratumoral section. **C)** Quantitative assessment revealed significant positive correlations between *in vivo* tumor and *ex vivo* brain SUV_mean_ (p < 0.001, Pearson's correlation). **D)** GZB density was found to be significantly increased in combination immunotherapy group relative to control (*, p < 0.05, unpaired two-tailed T test). **E)** Intratumoral GZB density was found to have significant positive correlations with SUV_mean_ TBR (R^2^ = 0.73, p < 0.01) and the tracer's dynamic binding rate (*k_3_,* R^2^= 0.83, p < 0.01)*.*

Dynamic [^64^Cu]-NOTA-GZP PET was validated via *ex vivo* PET imaging, followed by IF for GZB for biological validation (**Figure 4A-B**). A strong positive correlation was found between *in vivo* tumor and *ex vivo* brain [^64^Cu]-NOTA-GZP PET SUV_mean_ (R^2^ = 0.89, p < 0.001), providing evidence for the proper quantification of intratumoral GZB presence following dynamic acquisition (**Figure 4C**) while removing potential inflammatory activity from the surgical site, as observed in our previous CD8-targeted PET study in GBM models 9. Histological quantification of GZB density revealed significant increases of intratumoral effector cell molecule presence following combination immunotherapy (519.7 ± 252 / mm^2^) when compared to saline controls (132.2 ± 35.64 / mm^2^, p < 0.05). This metric was further correlated with intratumoral [^64^Cu]-NOTA-GZP SUV_mean_ TBR (R^2^ = 0.73, p < 0.01) and the tracer's binding rate (*k_3_*) derived from a 2TCM fitting (R^2^ = 0.83, p < 0.01), providing evidence for the proper biological characterization of tumor metrics from this immune-targeted PET imaging approach.

### Compartment modeling of dynamic [^64^Cu]-NOTA-GZP PET imaging revealed intratumoral changes following combination immunotherapy

**Figure 5:**
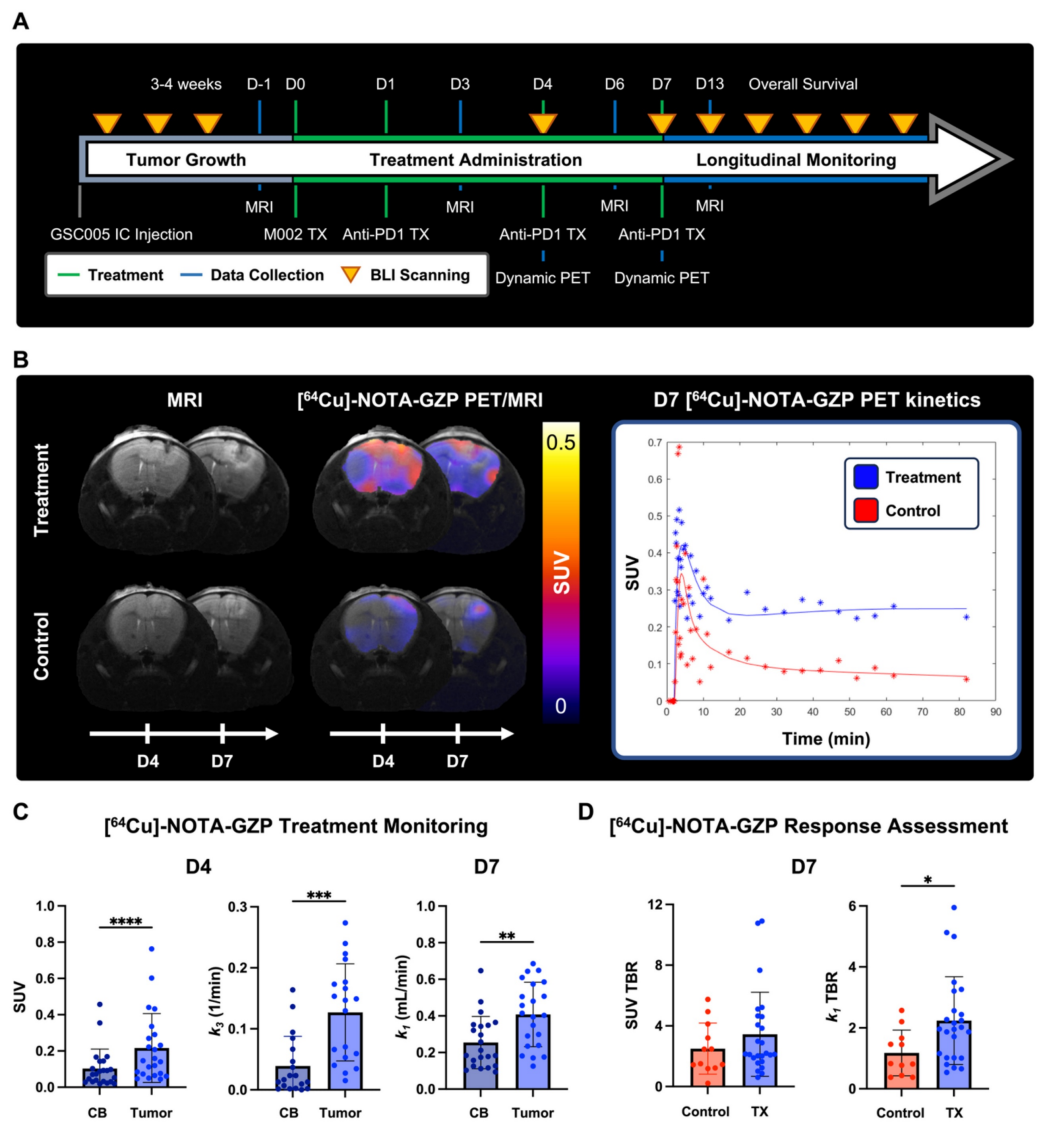
** Dynamic [^64^Cu]-NOTA-GZP PET quantification revealed intratumoral combination immunotherapy-induced changes. A)** Experimental timeline for longitudinal survival assessment with key treatment and imaging timepoints. **B)** Representative MRI and PET/MRI scans for treatment and control at days 4 and 7 following immunotherapy (left), with respective tracer kinetic curves at day 7 (right). **C)** Comparisons between contralateral brain (CB) and tumor region revealed significant increases in granzyme B presence and tracer binding at day 3 as well as increased tracer vascular transfer rate at day 7 in treated mice relative to controls (p < 0.001, paired two-tailed T test). **D)** Significant increases in tracer vascular transfer rate, as defined by *k_1_* TBR, were observed in treated mice compared to controls (p < 0.05, unpaired two-tailed T test) while trending increases were observed in tracer uptake as defined by SUV TBR (p > 0.05).

Quantitative [^64^Cu]-NOTA-GZP PET tracer kinetic properties were evaluated at 4- and 7-days following combination M002 and anti-PD1 ICB immunotherapies to assess changes in intratumoral properties (**Figure 5A-B**). Significant differences in intratumoral effector cell function and tracer binding rate, as defined by SUV (0.22 ± 0.19) 1 h post-injection and *k_3_* (0.13 ± 0.8 / min), were observed in combination immunotherapy-treated tumors four days following therapy relative to contralateral brain (SUV, 0.10 ± 0.11, p < 0.0001; *k_3_*, 0.04 ± 0.05 / min, p < 0.001). In addition, significant differences in intratumoral vascular tracer influx, as defined by *k_1_* (0.41 ± 0.18 mL / min) were observed seven days post-therapy relative to contralateral brain (0.25 ± 0.14 mL / min, p < 0.01), see **Figure 5C**. Of importance, the described differences in tracer kinetic metrics for tracer binding at day 3 and vascular influx at day 7 between tumor and contralateral brain were not observed in controls (p > 0.05, **Figure S3A**), while similar increases in intratumoral SUV at day 3 were observed in this cohort (p < 0.05). In addition, significant increases in intratumoral vascularity, as defined by *k_1_* TBR, were observed in the combination immunotherapy group (2.24 ± 1.44 mL / min) relative to controls (1.18 ± 0.75 mL / min, p < 0.05) at day 7, while trending increases were observed in SUV TBR between these groups (control, 2.50 ± 1.68; treatment, 3.45 ± 2.8; p > 0.05), see **Figure 5D**. These findings provide evidence for the complementary nature of dynamic PET imaging metrics, which can further inform on immunotherapy-induced changes within the tumor microenvironment.

### Longitudinal BLI and MRI monitoring allowed for the classification and radiological characterization of combination immunotherapy responses

**Figure 6:**
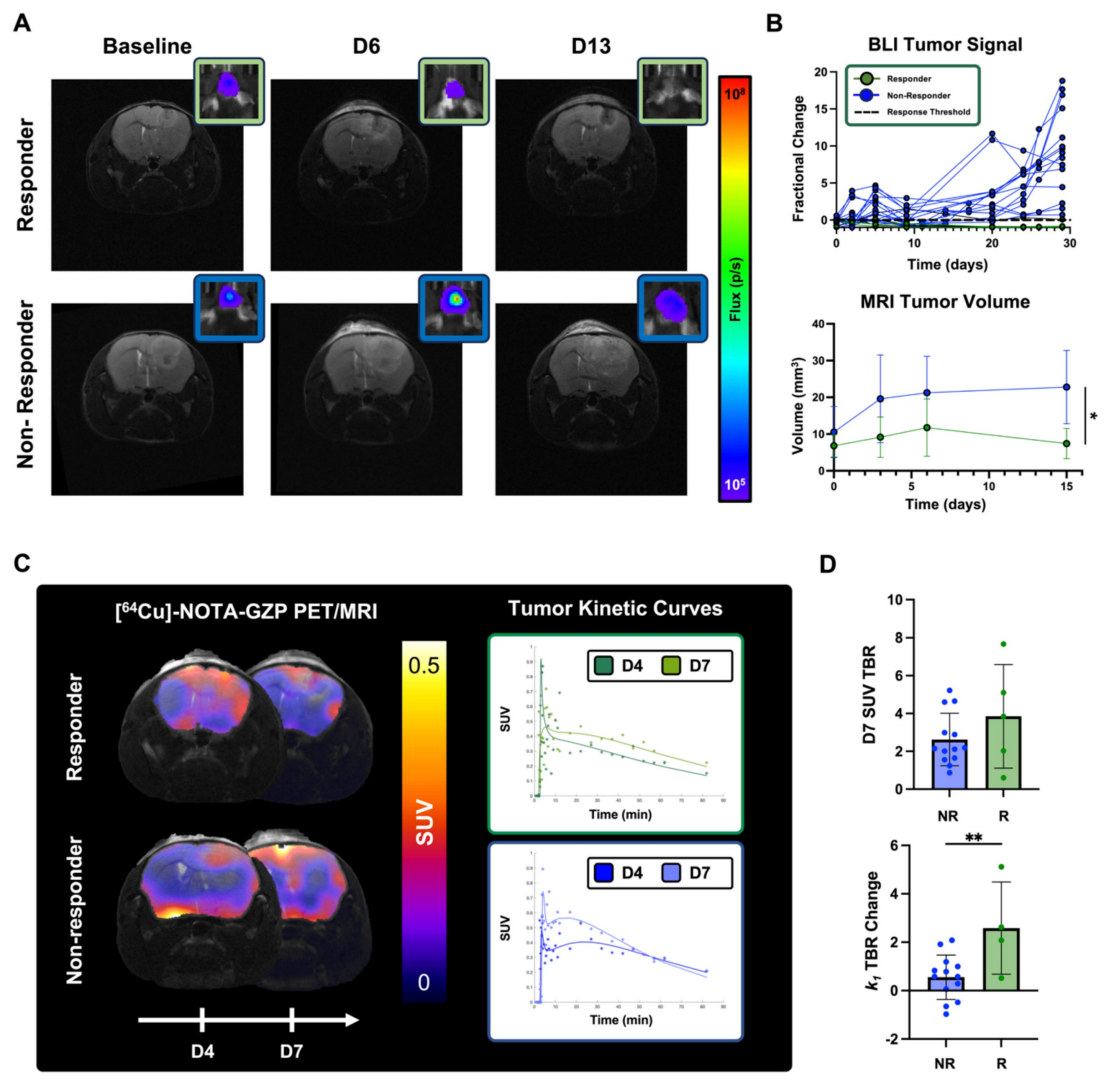
** Longitudinal [^64^Cu]-NOTA-GZP PET imaging provided metrics for the early characterization of M002 and anti-PD1 immunotherapy response. A)** Representative anatomical *T_2_*-weighted MRI and respective BLI scan for a combination immunotherapy responder and non-responder at baseline and at day 3, 6 and 13 post-therapy. **B)** Longitudinal monitoring of BLI allowed for the classification of combination immunotherapy responses one month following immunotherapy. Significant differences in tumor volumes, as defined from anatomical MRI, across treatment groups were observed two weeks following immunotherapy (p < 0.05, unpaired two-tailed T-test). **C)** Representative [^64^Cu]-NOTA-GZP PET scans and tumor kinetic curves for a combination immunotherapy responder and non-responders at days 4 and 7. **D)** Intratumoral dynamic [^64^Cu]-NOTA-GZP PET imaging quantification revealed significant differences in vascular changes, as defined by *k_1_* TBR, in responders relative to non-responders (p < 0.05), while no differences in effector cell function, as defined by SUV TBR, were observed across response groups (p > 0.05).

BLI and MRI allowed for the monitoring of changes in tumor cell viability and radiographic tumor volume following combination M002 and anti-PD1 (**Figure 6A**). Evaluation of BLI signal at two different days of dynamic [^64^Cu]-NOTA-GZP PET (days 4 and 7) showed no significant differences between responders and non-responders (p > 0.05). Response stratification based on BLI fractional changes at day 12 demonstrated a 0% response rate in the control group (N = 0 / 9) and 23% response rate in the combination immunotherapy group (N= 5 / 22). When evaluating tumor volumes as defined by anatomical MRI, no significant differences were observed between responders and non-responders at baseline, and three and seven days following M002 and anti-PD1 (p > 0.05), resembling anatomical MRI limitations observed in clinical immunotherapy studies. Follow-up MRI evaluation two weeks following therapy showed significant differences in tumor volumes between responders (7.42 ± 4.12 mm^3^) and non-responders (22.78 ± 9.97 mm^3^, p < 0.05), see **Figure 6B**. All mice classified as therapeutic responders at day 12, as defined by BLI, exhibited signs of recurrence within three months from the initiation of therapy, as defined by gradual increases in BLI signal (**Figure S4**).

### Longitudinal dynamic [^64^Cu]-NOTA-GZP imaging quantification revealed differences in intratumoral metrics in combination immunotherapy responders

Dynamic [^64^Cu]-NOTA-GZP PET/MRI imaging was performed at days 4 and 7 for the monitoring of intratumoral tracer kinetics and effector cell molecule changes following combination immunotherapy response (**Figure 6C**). Significant differences were observed in changes in tumor tracer perfusion across response groups, with responders exhibiting higher increases (2.59 ± 1.91) between day 4 and day 7 relate to non-responders (0.56 ± 0.92, p < 0.01). In addition, no significant differences were observed in effector cell function, as defined by [^64^Cu]-NOTA-GZP SUV TBR, between responders (3.85 ± 2.74) and non-responders (3.71 ± 3.16) one week following immunotherapy (p > 0.05), see** Figure 6D**.

## Discussion

In this study, we explored the use of compartment modeling to describe [^64^Cu]-NOTA-GZP PET kinetics and evaluated its association with response to novel combination immunotherapy in an orthotopic syngeneic GBM model. Dynamic imaging of immune-targeted PET tracers allows for the complementary characterization of immunological populations and tracer kinetics, providing a comprehensive assessment of immunotherapy-induced changes within the GBM tumor microenvironment. Initially, we demonstrated that tumoral [^64^Cu]-NOTA-GZP tracer kinetics were properly described by a two-tissue compartment model, in line with previously published literature on dynamic PET pharmacokinetic modeling for amino acid PET tracers in preclinical GBM studies 16,34,35. We histologically confirmed that combination immunotherapy resulted in an increased intratumoral expression of granzyme B, associated with enhanced antitumoral immune activity, which correlated with tracer binding kinetics and intratumoral uptake derived from dynamic [^64^Cu]-NOTA-GZP PET. Longitudinally, we found that immunotherapy responses showed associations with enhanced tracer vascular perfusion and increasing trends of granzyme B presence prior to measurable volumetric changes derived from anatomical MRI. In this contribution, we present, to our knowledge, the first evaluation of compartment modeling for dynamic granzyme B PET imaging to describe its pharmacokinetic characteristics in primary brain malignant lesions during novel immunotherapeutic approaches. This work integrates kinetic modeling for immune-targeted PET tracers and shows its first use as a complementary response assessment approach in preclinical orthotopic GBM models following immunotherapy.

Implementation of mathematical compartment models allows for the quantitative measurement of tracer pharmacokinetics informative on vascular delivery and tracer interactions, with previously demonstrated improvements in prognostic value in preclinical GBM models 34. Conventionally, dynamic PET imaging in preclinical brain tumors has been evaluated utilizing 2TCMs, descriptive of intratumoral perfusion and tracer binding, with agents targeting amino-acid transporters including [^18^F]FET and [^18^F]-fluciclovine, and molecular targets such as prostate specific membrane antigen (PSMA) small-molecule inhibitors 16,34. In our study, we considered the addition of a third component accounting for non-specific binding, as well as simplification to a single compartment as potential descriptors for the pharmacokinetics of an immune-targeted peptide-based tracer 20. Our comparative assessment on an immune-target PET agent showed similar suitability of a 2TCM to describe its pharmacokinetic properties in a GBM model. Furthermore, clinical studies have highlighted decreases in fitting variance and uncertainty when utilizing 2TCM over 3TCM for voxel-wise fitting of dynamic PET data 36, providing supportive evidence for its use in the description of [^64^Cu]-NOTA-GZP tracer pharmacokinetics. Conversely, 1TCM failed to properly capture the observed tracer retention, as defined by endpoint uptake, over the course of acquisition in comparison to 2TCM and 3TCM, suggesting the need to integrate additional compartments for the modeling of dynamic PET imaging data 28. Based on this assessment, we showed that 2TCM-based modeling provides an appropriate description of [^64^Cu]-NOTA-GZP tracer pharmacokinetics in preclinical orthotopic GBM models.

Combination immunotherapy consisting of IL-12-armed oHSV and immune checkpoint blockade has been shown to induce intratumoral immune infiltration, resulting in antitumoral immune effects in preclinical GBM models 8,9. We observed that combination immunotherapy resulted in increased cytotoxic cell activity and tracer binding four days following the start of therapy, demonstrating the early immunological benefit of this therapy. Since adaptive cytotoxic T cell activity commonly peaks one week following immunotherapy, this early increase in tracer binding and granzyme B presence could be reflective of innate immune activation, particularly NK cell recruitment and cytotoxic activity 37. This response can further be associated with the oHSV-mediated release of damage-associated molecular patterns (DAMP) and pathogen-associated molecular patterns (PAMP) following cancer cell lysis, resulting in innate immune promotion 5. Furthermore, in our histological analysis, we found significant associations between intratumoral granzyme B density, as measured by number of GZB^+^ sites per mm^2^, and the tracer binding rate (*k_3_*), derived from dynamic [^64^Cu]-NOTA-GZP PET. This finding can be attributed to increased peptide-specific binding over time when more biological target is present within the tumor. This metric can provide complementary information on GZB presence to tracer uptake quantification, which showed a similar positive correlation while being slightly weaker due to the potential influence of the inability of this tracer to bind to inactivated intracellular granzyme B, making it solely indicative of extracellular activation 21. These outcomes highlight the complementary benefit of dynamic PET-derived metrics from immune imaging as they can further describe intrinsic biological properties to improve the characterization of intratumoral changes associated with novel immunotherapy response 38.

Mathematical description of [^64^Cu]-NOTA-GZP tracer kinetics provided metrics associated with combination immunotherapy response, complementary to standard SUV assessment. In this cohort, we observed trending associations between higher SUV, indicative of increased effector cell function, and immunotherapy response one week following treatment administration. This non-invasive monitoring of downstream antitumoral immune function offers a unique approach to evaluate early immunotherapy response by directly targeting markers of therapy-induced activity, with previously demonstrated relevance for distinguishing downstream immunotherapy response in other cancer models 21,19. Furthermore, we observed that SUV as a singular metric was not able to clearly differentiate early immunotherapy responders in this model of glioma, characterized by its relatively cold immune tumor microenvironment. These findings resemble our previous assessment where CD8 cell spatial distribution was found to be informative of downstream decreases in tumor burden 9,39. Potential factors that could influence this inability of standard SUV to clearly differentiate responders could include enhanced permeability and retention (EPR) effects and non-specific binding confounding granzyme B quantification in GBM models 9,40. Nonetheless, our findings of increases in SUV in combination immunotherapy-treated mice relative to controls point towards an influence of immunotherapy-induced effector cell function despite remaining heterogeneous given the expected variability in therapy response. Furthermore, the use of dynamic acquisition protocols characterizing tracer vascular access and binding can further elucidate on these effects, potentially enhancing the characterization of the tumor microenvironment when assessing immunotherapy response.

By modeling tracer kinetics, we observed that combination immunotherapy resulted in increased [^64^Cu]-NOTA-GZP vascular transport rate (*k_1_*), descriptive of tracer influx from vasculature into the tumor space 41, one week post-treatment. In untreated orthotopic GBM models, this metric has been shown to be descriptive of tumor vascularization, influenced by angiogenesis, vascular apoptosis, permeability and blood-brain barrier leakage 16. Treatment with M002 oHSV was expected to result in anti-angiogenic effects given the vascular endothelial growth factor inhibitory effects attributed to the IL-12 therapeutic payload of this virotherapy 7,42,43. These antiangiogenic agents have the potential to induce transient tumor vessel normalization, improving perfusion, decreasing hypoxia and enhancing cytotoxic therapies 44. In immunotherapy, vascular normalization has further been shown to enhance immune cell infiltration and improve therapeutic responses, with immunotherapy-activated CD4^+^ T cells playing a key role in this promotion 45-47. In our study, we observed that temporal increases in *k_1_* were associated with combination immunotherapy response, which could be linked to vascular normalization allowing for improved cytotoxic immune cell infiltration and homogeneity in intratumoral distribution, collectively leading to enhanced treatment efficacy. These findings support the use of dynamic PET imaging-derived metrics to characterize complementary vascular properties with the potential to improve early assessment of immunotherapy response in GBM.

Anatomical MRI remains the gold standard for assessment of immunotherapy response in GBM 48, while clinical reports evaluating oHSVs have highlighted its limitations when differentiating tumor progression from inflammatory effects 10,11. Our *T_2_*-based MRI monitoring resembled these findings as responder and non-responder mice exhibited similar radiological tumor volumes early over the course of therapy, with a follow-up assessment being required for proper response stratification. In addition, upon therapeutic response, mice showed eventual recurrence within three months of observed curative effects, resembling the limited therapeutic benefit of single agent anti-PD1 and IL-12-armed oHSV previously shown in this model 8. As described in this study, dynamic [^64^Cu]-NOTA-GZP PET approaches can provide essential information for the stratification of patient response to immunotherapy at timepoints prior to measurable volumetric changes.

As an agent in clinical trials (NCT04169321), clinical translation of the proposed methodologies could be further extended to evaluate the prognostic value of mathematical modeling in dynamic [^64^Cu]-NOTA-GZP PET imaging. As a non-invasive imaging approach, integration of GZB-targeted PET imaging into therapeutic planning and response monitoring for immunotherapies has the potential to distinguish pseudoprogression from true tumor progression early over the course of therapy, serving as guidance for therapeutic management and clinical interventions to improve clinical outcomes for GBM. In addition, this PET approach could allow for the recognition of immunologically silent regions, providing guidance for tumor biopsies, resections and catheter placement sites for intratumorally infused therapies, such as oHSV, as well as allowing for the longitudinal monitoring of localized responses. Of importance, as an imaging approach targeting variable or potentially absent biomarkers within the GBM tumor microenvironment, dynamic [^64^Cu]-NOTA-GZP PET imaging would likely require complementary imaging capable of delineating tumor boundaries, such as *T_1_*+C MRI or amino acid PET, to accurately distinguish intratumoral effector cell function activity in a post-immunotherapy setting. Outside the central nervous system, as immunotherapy can be administered systemically, intratumorally or through both routes, as in this study, additional systemic increases in immune activation can be expected. Reports have demonstrated this in lymph nodes and spleen 9,14,49, where antitumoral immune responses are primed, and have also seen immune responses in healthy tissues which result in immune-related adverse events (irAE) 50. Due to its specificity to immune effector cell activity, this imaging approach has an opportunity to capture non-tumor immune kinetics and provide early information on the onset of irAEs. Furthermore, clinical acquisition of dynamic PET scans greater than 80 min are unlikely to be clinically feasible, where alternatives such as protocol simplification to an early infusion dynamic acquisition (20-30 min) paired with a late static scan (1-4 h following injection) could allow for the characterization of tracer kinetics and overall uptake with retained clinical relevance, while studies validating the quantitative accuracy of these simplified protocols would be required.

Despite these promising findings for the characterization of tracer kinetics and patterns of response in GBM immunotherapy with dynamic [^64^Cu]-NOTA-GZP PET, our study carries some limitations. As the primary goal of the study was to evaluate dynamic [^64^Cu]-NOTA-GZP PET-derived metrics for immunotherapy response assessment in GBM, we solely evaluated combination oHSV and anti-PD1 ICB as a treatment strategy given its improved response rates in GBM models, which have relatively cold immune tumor microenvironments, to ensure a balance of both responders and non-responders 8,9. While outside the scope of this study, the strategies derived from this evaluation can be translated to other treatments for GBM including single-agent ICB, oHSV or dendritic cell immunotherapies, where similar mechanisms of effector cell function and vascular accessibility play a relevant role in downstream response 9,20,21,51. For compartment modeling, we utilized an image-derived AIF extracted from the heart region, which could introduce variability when compared to direct blood sampling, population-based approaches or derivations from other vascular sites such as the inferior vena cava or carotid arteries 52-54. Image-derived individual AIFs were chosen as challenges can be introduced by complex invasive procedures during direct blood sampling, potential errors derived for intersubject variability in population-based AIFs, and susceptibility for partial volume effects when deriving AIFs from small vessels 52,53,55. Additionally, PET and MRI were collected on separate acquisition systems, requiring the use of landmark-based rigid registration methods. While the skull can serve as a reference structure for registration, previously described in other studies 23,24, there is potential for slight variations in alignment. In this context, effects were mitigated by evaluating whole tumor kinetics in contrast to voxel-wise approaches, where noise was reduced while sacrificing spatial resolution. This tradeoff was carefully considered for mouse models of glioma, that are relatively small in size, due to the spatial limitations of PET. Future studies in larger animal models and clinical trials could allow for voxel-level kinetic modeling where the collected spatial information can further help characterize localized responses, intratumoral heterogeneity, and therapy-induced changes within the GBM tumor microenvironment.

In conclusion, this study provides a biologically grounded mathematical framework for the mathematical description of dynamic [^64^Cu]-NOTA-GZP PET and demonstrated its utility in characterizing immunotherapy response in GBM. Our study focused on utilization of compartment modeling as a descriptor for tracer pharmacokinetics since it allows for the derivation of biologically interpretable quantitative metrics and provides flexibility to incorporate or simplify kinetic features. Tracer tumor pharmacokinetics were best described through a two-tissue compartment model and metrics showed associations with histological assessment. Immunotherapeutic responses were associated with increased intratumoral tracer transfer rate prior to changes in measurable tumor volume, providing a clinically translatable metric to identify early therapy response. Integration of these quantitative dynamic immune-targeted PET approaches has the potential to inform on key therapeutic-response patterns, allowing for the early stratification and prediction of patient outcomes following immunotherapy in GBM.

## Supplementary Material

Supplementary figures.

## Abbreviations

AIC: Akaike information criterion
AIF: arterial input function
BLI: bioluminescence imaging
CB: contralateral brain
Conc: concentration
CT: computed tomography
DAMP: damage-associated molecular pattern
EPR: enhanced permeability and retention
FA: flip angle
FDG: fluorodeoxyglucose
GBM: glioblastoma multiforme
GZB: granzyme B
GZP: granzyme B specific PET imaging agent
H&E: hematoxylin and eosin
HPLC: high-performance liquid chromatography
IACUC: Institutional Animal Care and Use Committee
ICB: immune checkpoint blockade
IF: immunofluorescence
IL: interleukin
irAE: immune-related adverse event
MRI: magnetic resonance imaging
NK: natural killer
NOTA: NOTA-Ala-Gly-Gly-Ile-Glu-Phe-Asp-CHO
oHSV: oncolytic herpes simplex virus
PAMP: pathogen-associated molecular pattern
PD1: programmed cell death 1
PET: positron emission tomography
PSMA: prostate specific membrane antigen
RARE: rapid acquisition with refocusing echoes
RM: repeated measures
ROI: region of interest
SUV: standardized uptake value
TBR: tumor to background ratio
TCM: tissue compartment model
TE: echo time
TR: repetition time
UAB: University of Alabama at Birmingham
