# Supplementary material for: Quantitative dynamic granzyme B PET imaging to characterize novel combination immunotherapy response in preclinical glioblastoma models
Source: Theranostics. 2026 Jan 1;16(6):2649–64. doi: 10.7150/thno.123588 (PMC12775653; doi:10.7150/thno.123588)
Supplement: Supplementary file 1 — Supplementary figures. [file thnov16p2649s1.pdf]

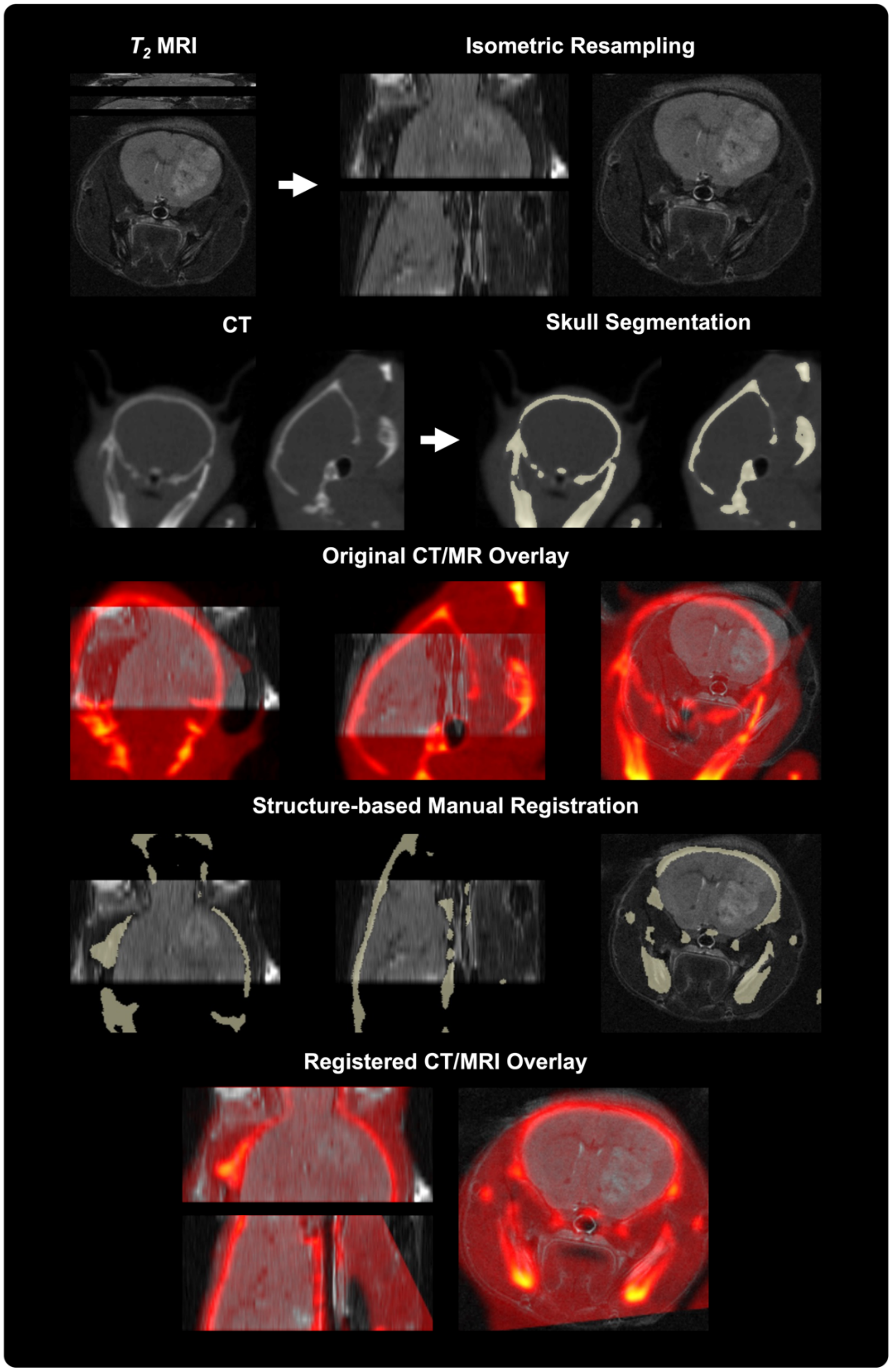

Figure S1. Representative schematic for CT/MRI registration workflow. The T_2 MRI scan was linearly resampled to its axial resolution, generating an isometric scan. The CT

scan was then resampled to match this resolution and used to generate a bone mask via optimal thresholding. This bone mask served as the reference for manual rigidly registration of the MRI and CT scans, using skull structures as anatomical landmarks. The dynamic PET scan was subsequently registered using the same transformation matrix. Representative aligned CT/MRI following this registration approach is shown at the bottom.

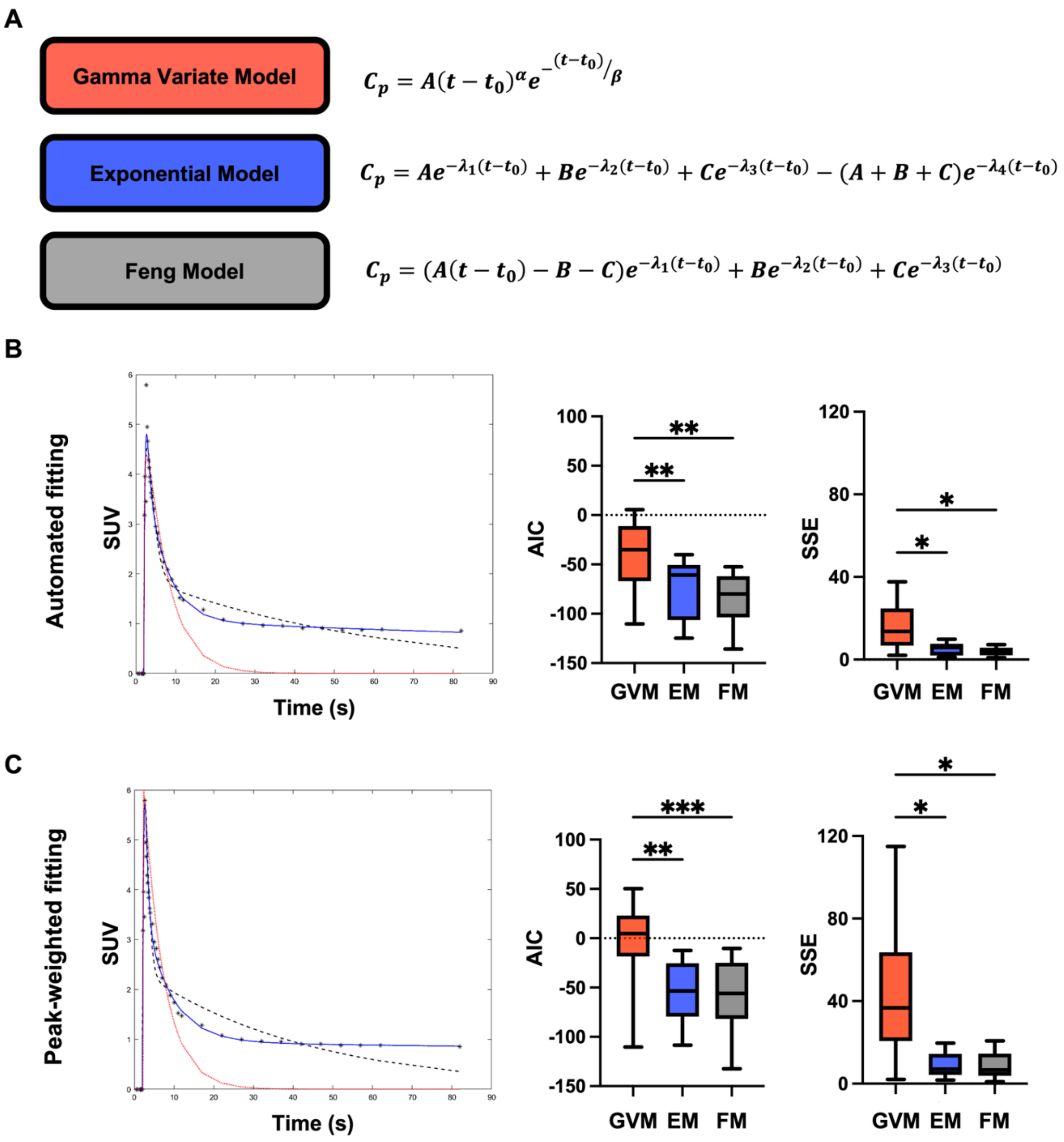

Figure S2. Arterial input function fitting comparative assessment. A) Three models (gamma variate, exponential and Feng) were evaluated for the description of the dynamic [^{64}Cu]-NOTA-GZP PET heart time activity curve. **B)** Representative plot of AIF automated fitting based on the evaluated models. Significant decreases in fitting quality, as represented by increases on Akaike Information Criterion (AIC) Sum of Square Errors (SSE), were observed based on gamma variate model relative to the exponential and Feng models (AIC, $p< 0.01$; SSE, $p> 0.05$). **C)** Evaluation

introducing increased penalty for the maximum value (weight= 10) was performed to ensure proper representation of tracer plasma kinetics. Similar findings were observed with decreased fitting quality when utilizing a gamma variate model (AIC, $p < 0.01$; SSE, $p > 0.05$), while no differences were observed between Feng and exponential models.

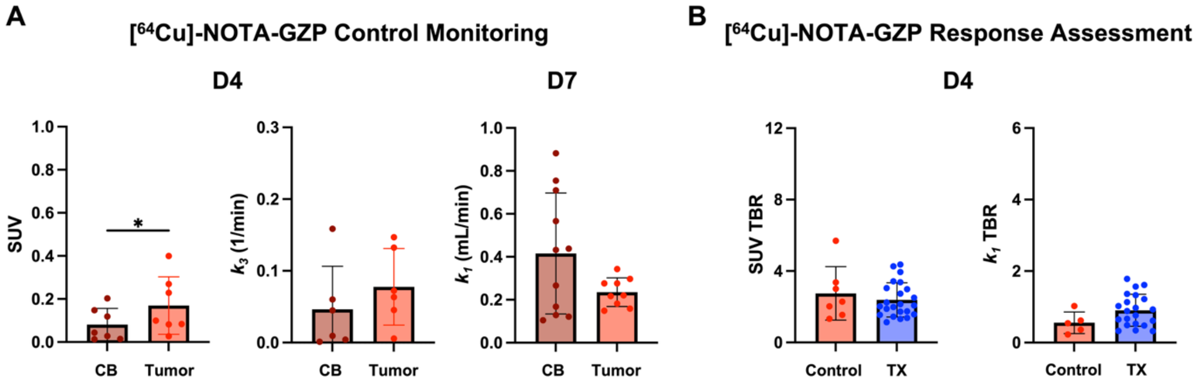

Figure S3. Complementary dynamic [^{64}Cu]-NOTA-GZP PET quantification results. A) Comparisons between MRI-defined tumor region and contralateral brain in control mice revealed an absence of differences in tracer kinetic metrics for tracer binding at D3 and perfusion at D7 ($p > 0.05$). In contrast, significant differences were observed in granzyme B presence, as defined by SUV, between tumor and normal brain at D3 ($p < 0.05$). **B)** Comparisons between treatment groups revealed no significant differences between controls and responders when evaluating effector cell function and tracer vascular influx ad D4 ($p < 0.05$).

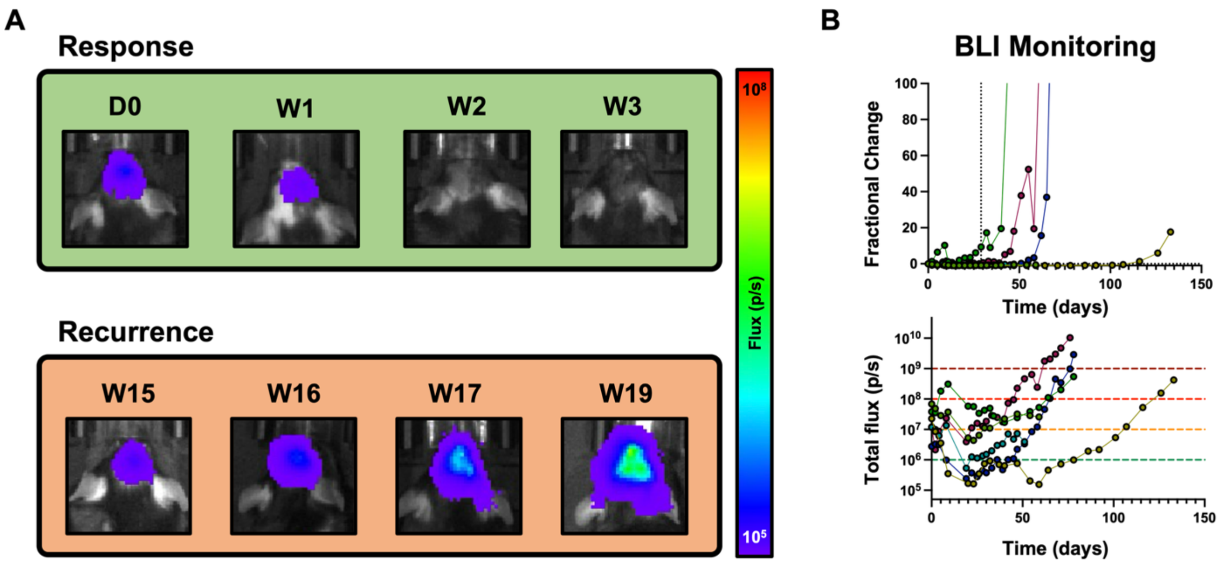

Figure S4. Tumor recurrence was observed in mice showing initial response to combination M002 and anti-PD1 immunotherapy. A) Representative BLI scans of responder mouse exhibiting early decreases in tumor burden (top) followed by subsequent downstream tumor recurrence (bottom). **B)** Increases in BLI signal, characteristic of recurrence, were observed as early as one month following positive response in all mice classified as responders, resembling clinical findings of GBM following immunotherapy.
